# Association Between Exercise Behavior Stages and Obesity Transition in Children and Adolescents: A Nationwide Follow-Up Study

**DOI:** 10.3390/nu17162608

**Published:** 2025-08-11

**Authors:** Ziyue Sun, Jiajia Dang, Shan Cai, Yunfei Liu, Di Shi, Jiaxin Li, Yihang Zhang, Ziyue Chen, Tianyu Huang, Yang Yang, Peijin Hu, Jun Ma, Tianjiao Chen, Yi Song

**Affiliations:** 1 Institute of Child and Adolescent Health, School of Public Health, Peking University, Beijing 100191, China; 2 National Health Commission Key Laboratory of Reproductive Health, Beijing 100191, China

**Keywords:** exercise, physical activity, overweight, obesity, transtheoretical model

## Abstract

**Backgrounds:** To examine the association between stages of exercise behavior change, as defined by the transtheoretical model (TTM), and obesity progression among Chinese children and adolescents, with attention to gender and urban–rural differences. **Methods:** A total of 5006 Chinese children and adolescents aged 9–18 years were assessed in 2019 and followed up in 2020. Participants were categorized into five TTM stages: precontemplation, contemplation, preparation, action, and maintenance. Logistic regression models evaluated the associations between the TTM stages and obesity outcomes, including incident obesity and transitions from normal or overweight to obesity. Analyses were stratified by gender and urban–rural residence, and interaction effects were tested. **Results:** Compared to the maintenance stage, precontemplation (OR = 2.08, 95% CI: 1.45–2.99) and contemplation (OR = 1.48, 95% CI: 1.05–2.08) stages had higher obesity risk, with similar trends in follow-up incident obesity (precontemplation: OR = 1.63, 95% CI: 1.17–2.28; contemplation: OR = 1.47, 95% CI: 1.10–1.98). These associations were more pronounced among boys and rural residents. Significant interactions were observed between TTM stages, sex (*p* = 0.029), and residence (*p* = 0.005) in obesity transition. **Conclusions:** Exercise behavior stages are associated with obesity progression, particularly among boys and rural children. These findings underscore the importance of stage-specific interventions tailored to individual readiness for behavior change and contextual factors.

## 1. Introduction

Childhood and adolescent obesity is a growing public health concern worldwide [1]. The prevalence of child and adolescent obesity remains high and continues to rise in low-income and middle-income countries, with significant implications for both current and future health outcomes [2]. Early intervention is crucial to prevent obesity and promote healthier trajectories into adulthood. Physical activity plays a pivotal role in the prevention and management of obesity [3]. Regular exercise helps in maintaining a healthy weight, improving metabolic health, and reducing the risk of developing obesity-related complications [4]. Despite its benefits, encouraging sustained exercise behavior in children and adolescents presents considerable challenges.

The transtheoretical model (TTM) offers a valuable framework for understanding the stages of behavior change related to exercise [5,6,7]. TTM categorizes individuals into five stages: precontemplation, contemplation, preparation, action, and maintenance. Each stage reflects a different level of readiness to engage in and maintain regular physical activity [8,9,10]. By identifying the stage of exercise behavior, tailored interventions can be developed to effectively promote progression to higher stages of readiness and sustained behavior change [11,12].

TTM is particularly advantageous because it recognizes that behavior change is a process rather than a single event [13]. This model allows for the design of interventions that are stage-specific, thereby increasing the likelihood of success [14]. TTM has been applied to various health behaviors, including smoking cessation, dietary changes, and physical activity, with considerable success in different populations [15,16,17]. The ability to target interventions based on an individual’s readiness to change makes TTM a flexible and effective tool in public health strategies. Previous studies using TTM demonstrated varied distributions across the stages in different populations and contexts [18]. In the context of exercise behavior, a significant portion of individuals often falls within the precontemplation and contemplation stages, indicating a lack of readiness to initiate physical activity [18]. In Western countries, TTM has been widely used to design and evaluate health interventions, but there is a lack of extensive research on its application in Chinese children and adolescents. Understanding the distribution of TTM stages in this population can provide insights into their readiness for behavior change and inform the development of culturally tailored interventions.

The application of the TTM in Chinese children and adolescents is particularly relevant given the unique cultural, social, and environmental factors influencing exercise behavior and obesity awareness in this population. Previous studies suggested that adolescents in different cultural settings, such as Brazil, show gender-based differences in weight-related attitudes, with boys often lacking awareness about the importance of weight management and girls more frequently reporting dieting intentions [19]. In China, similar disparities are likely influenced by varying sociocultural expectations and access to resources. Urban–rural differences in physical activity opportunities and obesity awareness are also pronounced, with urban adolescents generally having better access to recreational facilities and health information, while rural youths may face more structural and socioeconomic barriers to healthy lifestyle behavior [20,21].

Despite growing interest in TTM theories, research examining the association between TTM stages and obesity progression remains limited, particularly among children and adolescents. Most of the existing studies have been cross-sectional and have not adequately captured the dynamic nature of obesity development in relation to motivational readiness for behavior change. Our study addresses this gap by investigating the prospective relationship between TTM-defined stages of exercise behavior change and obesity outcomes in a nationally representative cohort of Chinese children and adolescents.

Prior studies showed gender-specific patterns in exercise motivation and body image, where men may be less aware or concerned about weight-related issues, while women may experience more pressure to be thin [22]. Urban–rural disparities in physical activity environments, access to resources, and health education are well-documented in China, potentially leading to different patterns of behavior change readiness and obesity risk [23,24,25]. Based on previous literature demonstrating that low intention to engage in physical activity is associated with increased sedentary behavior and a higher risk of weight gain among adolescents [26,27], we hypothesized that children and adolescents in the precontemplation stage—characterized by low motivation or intention to engage in physical activity—would have higher obesity incidence and greater risk of transitioning to obesity compared to those in the maintenance stage. Based on the 2019 baseline data, we classified participants into stages of exercise behavior and assessed their initial weight status. The 2020 follow-up data enabled us to track weight status changes over time, including new-onset and transitions in obesity. This longitudinal design aimed to investigate both cross-sectional and prospective associations between TTM stages and obesity transition and to explore how these stages are associated with the incidence and transition of obesity, with a focus on potential differences by sex and urban–rural residence.

## 2. Materials and Methods

### 2.1. Study Design and Participants

**Figure 1:**
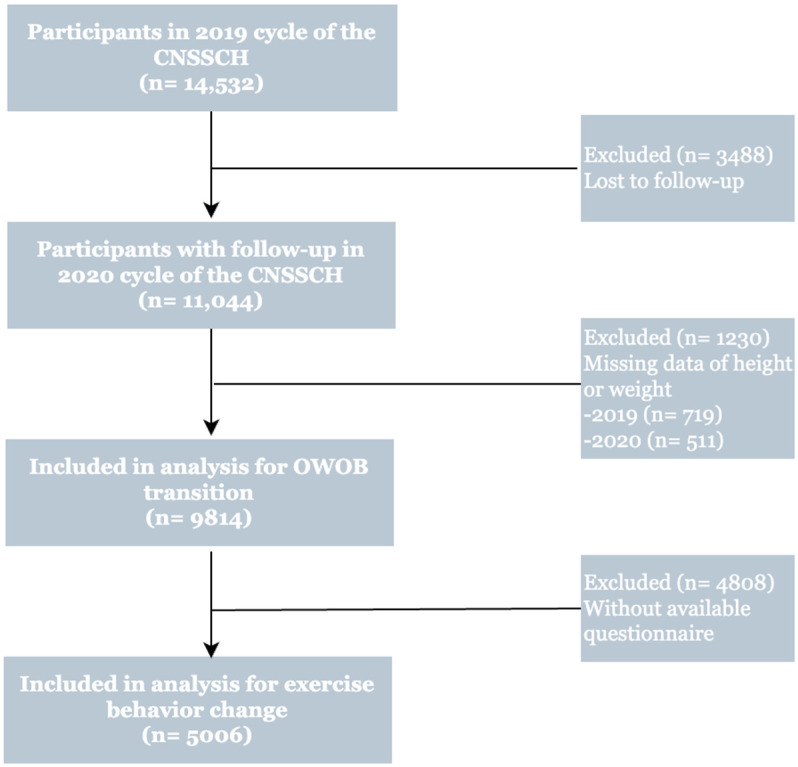
Study flow.

Data from the 2019 cycle of the Chinese National Survey on Students’ Constitution and Health (CNSSCH) served as the baseline [21]. Eight provinces were randomly chosen across eastern, central, and western geographical regions for a follow-up survey conducted in November 2020. The participants were taken from eight provinces representing eastern, central, and western China, ensuring regional diversity. The sample, therefore, reflects a nationally representative cohort of school-aged children and adolescents in China, with consideration of geographical, socioeconomic, and demographic diversity. The baseline dataset initially included 14,532 children and adolescents aged 6–18 years (shown in Figure 1). For descriptive analysis, 9814 individuals within the same age range were retained. Given that the questionnaire was only administered to those aged 9 years and older, the association analysis focused on 5006 participants in the 9–18-year age group. This excluded 6–8-year-olds who did not complete the questionnaire and older children with missing key questionnaire data (e.g., physical activity-related information). Informed consent was obtained from all participants and their guardians, and the study was approved by the Medical Research Ethics Committee of Peking University Health Science Center (IRB00001052-18002, IRB00001052-21001).

### 2.2. Overweight (OW) and Obesity (OB) Measurement

Trained technicians followed standardized protocols to measure height and weight. Participants were asked to wear lightweight clothing and remove footwear during measurements, which were recorded to the nearest 0.1 cm and 0.1 kg, respectively. Body mass index (BMI) was calculated as weight (kg) divided by height (m) squared. Overweight and obesity (OWOB) were categorized based on sex- and age-specific percentiles defined by China’s National Health Commission [28]: OW was defined as BMI ≥ 85th percentile, and OB as BMI ≥ 95th percentile. Transition to obesity was defined as participants with normal weight, thinness, or overweight at baseline developing obesity (OB) during follow-up.

### 2.3. Definition of the Stage of Change for Exercise Behavior Based on TTM

The stage of change for exercise behavior based on the TTM are described in Table 1. The measures [29,30,31] had been translated into Mandarin and successfully used in prior studies [32,33,34]. The stage of change for exercise behavior was assessed using the algorithm approach recommended by Reed and colleagues [23]. On the measure, exercise was defined as engaging in behaviors such as walking, jogging, swimming, biking, playing basketball, table tennis, badminton, or aerobic dance three or more times per week for 30 min or more each time. The specific questions include “I am currently actively participating in physical exercise” (Q1), “I will participate more actively in physical activities in the next six months” (Q2), “I currently maintain regular physical activity” (Q3), and “I have been engaging in regular physical activity for the past six months” (Q4, with options 0 = No, 1 = Yes), and based on the responses to these questions, the participants were classified into five stages: precontemplation, contemplation, preparation, action and maintenance stage. The precontemplation stage refers to the situation where participants do not plan to start exercising, with the criterion being Q1 = 0 and Q2 = 0. The contemplation stage refers to the situation where participants plan to start in the next 6 months, with the criterion being Q1 = 0 and Q2 = 1. The preparation stage refers to the situation where participants are planning to start in the next 30 days, with the criterion being Q1 = 1 and Q3 = 0. The action stage refers to the situation where participants have been exercising for less than 6 months, with the criterion being Q1 = 1, Q3 = 1, and Q4 = 0. The maintenance stage refers to the situation where participants have been exercising for more than 6 months, with the criterion being Q1 = 1, Q3 = 1, and Q4 = 1. The TTM measures showed good internal consistency of the scale items (Cronbach’s α range = 0.60–0.89), indicating acceptable reliability.

### 2.4. Questionnaire Survey

Trained fieldworkers administered paper-based questionnaires to 9–18-year-olds during class time. Participants filled out the questionnaires independently, with researchers providing relevant support when needed. The survey collected sociodemographic characteristics (age, sex, residence, grade, single-child status, parental education level), lifestyle factors (sugar-sweetened beverage intake, sleep duration), and exercise behavior. The survey demonstrated moderate internal consistency, with a Cronbach’s alpha of 0.76 for school-level factors [35].

### 2.5. Statistical Analysis

Missing data were handled using hot-deck imputation [36,37]. Chi-square tests were employed to assess sex and residential differences, while Mantel–Haenszel tests for linear trends evaluated age-group trends. Mixed-effects logistic regression models were utilized to estimate adjusted odds ratios (ORs) and 95% confidence intervals (CIs) for associations between behavioral stages and OW/OB outcomes, including transition to overweight or obesity. Multicollinearity was checked via variance inflation factors (VIF), confirming no variables had elevated VIF values. The adjusted model controlled for fixed effects (age, sex, residence, single-child status, breakfast frequency, sugar-sweetened beverage intake, sleep duration, parental education level) and random effects (provinces), incorporating province clustering as a random intercept to account for data hierarchy. Analyses were performed using SPSS 26.0 and R 4.2.2, with two-tailed *p* < 0.05 considered significant. Sensitivity analyses were conducted using the International Obesity Task Force (IOTF) criteria [34] to define OWOB, a globally accepted standard for pediatric weight classification, to validate results robustness.

## 3. Results

### 3.1. Overweight and Obesity Changes During the Follow-Up

**Figure 2:**
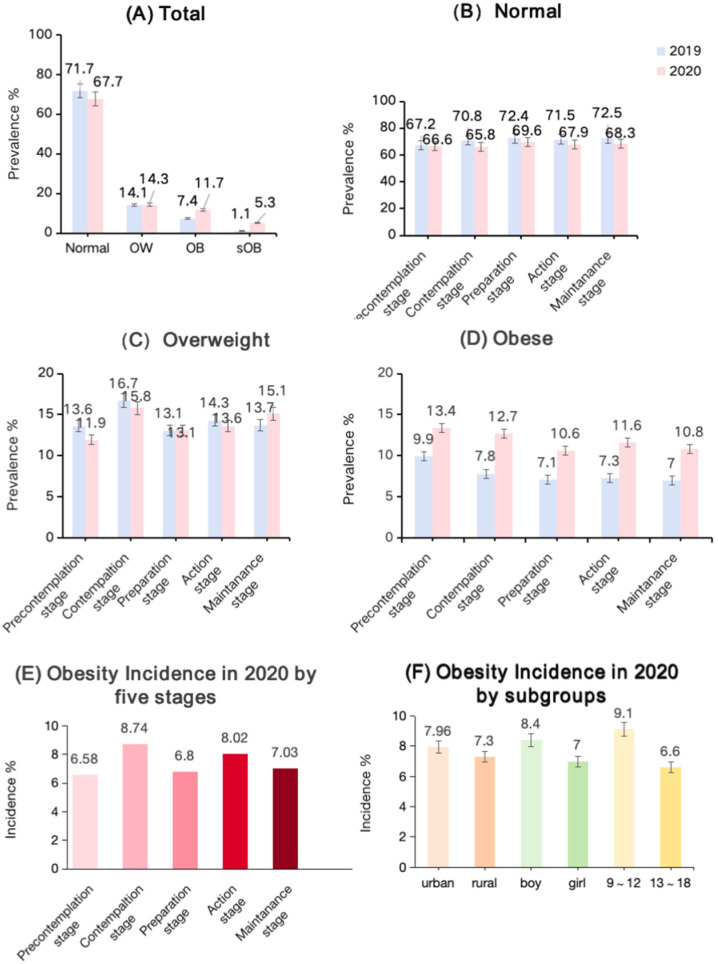
Prevalence, incidence of OWOB among children and adolescents by exercise behavior stages, residence, gender, and age groups. (**A**) Total prevalence; (**B**) Normal condition; (**C**) OW prevalence by exercise behavior stages; (**D**) OB prevalence by exercise behavior stages; (**E**) OB incidence by exercise behavior stages in 2020; (**F**) OB incidence by residence, sex, age in 2020. Each bar reflects the mean prevalence across provinces, and the error bar indicates standard deviation.

Table 2 shows the characteristics of eligible children and adolescents aged 9–18 years, stratified by gender groups. In our nationwide cohort study (n = 5006), the overall prevalence of normal weight declined from 71.7% at baseline (2019) to 67.7% at one-year follow-up (2020), while the proportions of overweight (14.1% to 14.3%), obesity (7.4% to 11.7%), and severe obesity (1.1% to 5.3%) all increased (shown in Figure 2A). When stratified by the exercise behavior stage, the prevalence of normal weight was highest in the maintenance stage (72.5% in 2019 vs. 68.3% in 2020) and lowest in the precontemplation stage (67.2% vs. 66.6%) (shown in Figure 2B). Overweight peaked in the contemplation stage (16.7% vs. 15.8%) (shown in Figure 2C) and obesity prevalence increased least in the preparation stage (7.1% to 10.6%) (shown in Figure 2D).

Obesity incidence over the one-year follow-up differed markedly by TTM stage: children in the contemplation stage (8.74%) and action (8.02%) stage experienced the highest new-onset obesity, compared with 6.58% in the precontemplation stage, 6.80% in the preparation stage and 7.03% in the maintenance stage (shown in Figure 2E). In the subgroup analyses, obesity incidence was slightly higher among urban versus rural participants (7.96% vs. 7.30%), greater in boys than girls (8.40% vs. 7.00%), and highest in the 9–12-year age-group compared with adolescents aged 13–18 years (9.10% vs. 6.60%) (shown in Figure 2F).

### 3.2. Association Between Stages of Exercise Behavior Change and Risk of OWOB in 2019

**Figure 3:**
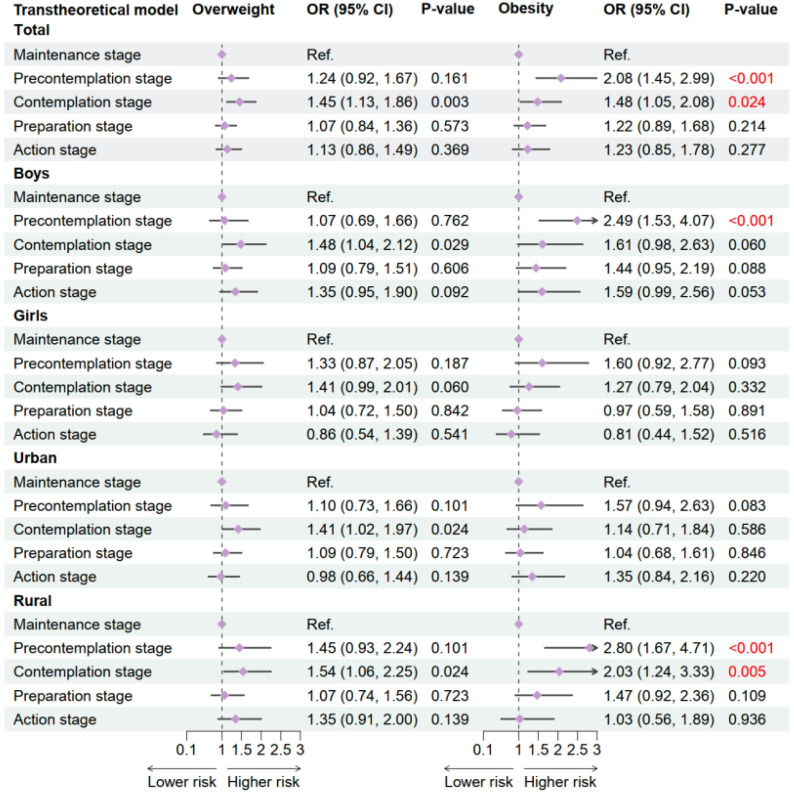
Association between exercise behavior stages and OWOB in 2019. Adjusted for age, sex, residence, single-child status, breakfast frequency, sugar sweetened beverage intake, sleeping duration, parental education level, and the clustered effect of provinces. OW, overweight; OB, obesity.

In multivariable logistic regression models using the maintenance stage as reference, significant associations were observed between the TTM stages and the likelihood of overweight and obesity in 2019 (shown in Figure 3). At the total population level, compared to the maintenance stage, individuals in the contemplation stage had significantly higher odds of both overweight (OR = 1.45, 95% CI: 1.13–1.86, *p* = 0.003) and obesity (OR = 1.48, 95% CI: 1.05–2.08, *p* = 0.024). The precontemplation stage was associated with more than double the risk of obesity (OR = 2.08, 95% CI: 1.45–2.99, *p* < 0.001), though its association with overweight was not statistically significant.

Subgroup analyses revealed sex- and region-specific patterns. Among boys, the contemplation stage significantly increased the risk of overweight (OR = 1.48, 95% CI: 1.04–2.12, *p* = 0.029), while the precontemplation stage was strongly associated with obesity (OR = 2.49, 95% CI: 1.53–4.07, *p* < 0.001). In contrast, no statistically significant associations were observed among girls. In urban children, contemplation was associated with increased odds of overweight (OR = 1.41, 95% CI: 1.02–1.97, *p* = 0.024), while obesity risk did not differ significantly across stages. Among rural participants, both the precontemplation (OR = 2.80, 95% CI: 1.67–4.71, *p* < 0.001) and contemplation stages (OR = 2.03, 95% CI: 1.24–3.33, *p* = 0.005) were significantly associated with higher obesity risk. These findings indicate that early stages of exercise behavior change, particularly precontemplation and contemplation, are strongly associated with increased obesity risk, especially in boys and rural children.

### 3.3. Association Between Stages of Exercise Behavior Change and OWOB Transition During the Follow-Up

**Figure 4:**
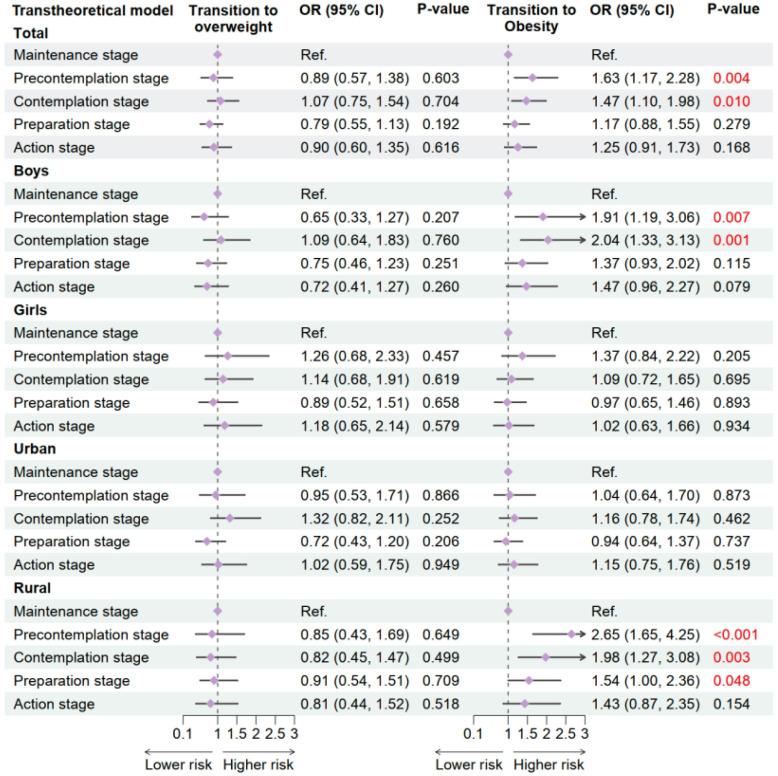
Association between exercise behavior stages and OWOB in 2019–2020 follow-up study. Adjusted for age, sex, residence, single-child status, breakfast frequency, sugar sweetened beverage intake, sleeping duration, parental education level, and the clustered effect of provinces. OW, overweight; OB, obesity.

Results from longitudinal analysis in 2020 showed that stages of exercise behavior based on the transtheoretical model were significantly associated with the transition to obesity, but not with the transition to overweight (shown in Figure 4). Using the maintenance stage as the reference, participants in the precontemplation stage had a significantly increased risk of transitioning to obesity (OR = 1.63, 95% CI: 1.17–2.28, *p* = 0.004), as did those in the contemplation stage (OR = 1.47, 95% CI: 1.10–1.98, *p* = 0.010). No statistically significant associations were observed for transition to overweight across all stages in the total population.

Subgroup analyses revealed that the relationship between early exercise stages and obesity transition was particularly pronounced among boys and rural children. Boys in the precontemplation stage (OR = 1.91, 95% CI: 1.19–3.06, *p* = 0.007) and contemplation stage (OR = 2.04, 95% CI: 1.33–3.13, *p* = 0.001) had significantly higher odds of becoming obese compared to those in the maintenance stage. Among rural participants, the precontemplation stage was associated with the highest obesity risk (OR = 2.65, 95% CI: 1.65–4.25, *p* < 0.001), followed by the contemplation (OR = 1.98, 95% CI: 1.27–3.08, *p* = 0.003) and preparation stages (OR = 1.54, 95% CI: 1.00–2.36, *p* = 0.048). These findings suggest that children and adolescents in earlier stages of exercise behavior change, especially those not yet engaged in regular physical activity, are at increased risk of developing obesity, particularly in boys and rural populations.

### 3.4. Interaction Effects of Exercise Behavior Stages with Gender and Region on OWOB Transitions During the Follow-Up

**Figure 5:**
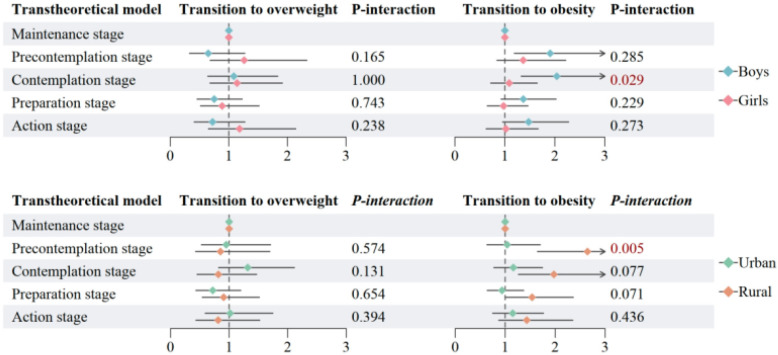
Interaction effects of exercise behavior stages with gender and region on OWOB transitions in 2020. Adjusted for age, sex, residence, single-child status, breakfast frequency, sugar sweetened beverage intake, sleeping duration, parental education level, and the clustered effect of provinces. OW, overweight; OB, obesity.

Figure 5 presents the interaction effects between stages of exercise behavior and gender or region on the transitions to overweight and obesity in 2020. A significant interaction between gender and the contemplation stage was observed for the transition to obesity (P-interaction = 0.029), indicating that the effect of being in the contemplation stage differed significantly between boys and girls. Specifically, girls in the contemplation stage had a higher risk of transitioning to obesity compared to boys in the same stage.

Additionally, a significant interaction was found between region and the precontemplation stage for the transition to obesity (P-interaction = 0.005), suggesting that residing in rural versus urban areas modifies the association between early exercise behavior stages and obesity risk. Rural participants in the precontemplation stage were more likely to become obese compared to their urban counterparts.

No significant interactions were detected for the transition to overweight, suggesting that gender and region may not have substantially modified the association between exercise behavior stages and overweight status within the one-year follow-up period.

## 4. Discussion

In this nationwide follow-up study of Chinese children and adolescents, we observed a marked shift in weight status between 2019 and 2020, with the prevalence of normal weight declining by 4%, accompanied by increases in overweight, obesity, and severe obesity. Importantly, the stages of exercise behavior change defined by the transtheoretical model (TTM) were differentially associated with both cross-sectional and longitudinal obesity outcomes. Participants in the precontemplation and contemplation stages consistently exhibited higher odds of prevalent obesity in 2019, incident obesity in 2020, and transitioning to obesity over the follow-up period compared to those in the maintenance stage. These associations were particularly pronounced among boys and rural residents.

Our study aimed to explore the association between TTM-defined stages of exercise behavior change and obesity outcomes among Chinese children and adolescents. We found that the distribution of TTM stages remained relatively stable across the two-year period, with most participants in the action stage. However, significant differences in the rates of transition to OW and OB were observed by gender and residence. Children in the precontemplation and contemplation stages faced notably higher risks of transitioning to obesity, especially boys and rural residents. These findings build upon previous cross-sectional research by providing prospective evidence that children with minimal or ambivalent motivation toward physical activity (i.e., those in the early TTM stages) are not only more likely to be obese at baseline, but are also at elevated risk of excessive weight gain over time.

Consistent with prior studies [38,39,40], our results highlight the critical role of the precontemplation and contemplation stages in shaping obesity trajectories. These early stages reflect a lack of readiness or intention to engage in regular physical activity, which likely contributes to higher obesity incidence. Multiple mechanisms may explain this association. Psychologically, children in these stages may lack motivation, awareness, or confidence regarding physical activity, leading to more sedentary lifestyles. Body image concerns may lead girls to avoid certain physical activities (e.g., team sports) due to self-consciousness [41]. Moreover, theories such as the self-determination theory emphasize that intrinsic motivation is essential for sustained behavior change [42], which is typically absent in early TTM stages. Biologically, reduced physical activity is known to lower energy expenditure and impair metabolic health, including decreased insulin sensitivity, which may elevate circulating insulin levels and promote fat accumulation [43,44]. Prior evidence has linked lower physical activity with increased insulin resistance, a precursor to type 2 diabetes and a key contributor to pediatric obesity [45]. Hormonal changes during puberty may further exacerbate obesity risk, especially in physically inactive adolescents [46]. Additionally, hormonal changes during puberty (e.g., estrogen fluctuations) may alter fat distribution patterns, making weight management more sensitive to non-exercise factors [47]. Social and environmental influences also play a role. Psychosocial stressors, such as academic pressure and social expectations, may also disproportionately reduce adolescents’ exercise adherence [48,49]. Children in the precontemplation stage may receive less peer or familial support for active behaviors, increasing their susceptibility to obesity [50].

Gender differences in our results were notable. Boys had higher overall rates of transitioning to both overweight and obesity compared to girls. Specifically, boys in the precontemplation and contemplation stages were at significantly greater risk of becoming obese, while such associations were not observed in girls. In the context of Chinese society, traditional gender norms may shape divergent attitudes and behaviors toward physical activity and weight management. Boys often receive less social and familial emphasis on weight control, which may result in lower awareness of the health risks associated with obesity [51]. Consequently, boys in the precontemplation or contemplation stages may lack intrinsic motivation to engage in physical activity, thereby exhibiting a stronger link between low readiness for exercise and subsequent obesity risk. In contrast, girls may be more likely to adopt non-exercise-based weight control strategies, such as dietary restriction or body image regulation [52], which could weaken the direct association between exercise behavior stages and obesity transition. Furthermore, psychological factors—such as greater concern about body image and social appearance—may influence girls’ weight-related behaviors independently of their physical activity readiness [53]. Additionally, physiological factors like hormonal fluctuations during puberty (e.g., elevated testosterone levels in boys) may exacerbate fat accumulation in the presence of physical inactivity, further amplifying obesity risk among inactive boys in early behavioral stages [54]. These gender-specific psychosocial and biological dynamics may help explain the observed gender differences in obesity transition patterns across the TTM stages. These gender-specific findings suggest that tailored interventions are warranted. For boys, strategies should focus on overcoming motivational barriers at earlier stages of behavior change. In contrast, although girls had relatively high transition rates to overweight and obesity, their obesity risk was not significantly modified by the TTM stages, suggesting that other factors, such as body image concerns, hormonal influences, or psychosocial stressors, may play more substantial roles [55,56].

Urban–rural disparities also emerged as important modifiers of the association between exercise behavior stages and obesity. While urban residents had higher overall transition rates to both overweight and obesity, rural children in the precontemplation and contemplation stages showed significantly elevated odds of transitioning to obesity compared to their urban counterparts. These results indicate that both urban and rural environments present distinct challenges to maintaining healthy behaviors. Rural children in China often face limited access to recreational infrastructure, lower health literacy, fewer organized physical activity opportunities, and greater socioeconomic constraints compared to their urban counterparts [57,58]. Meanwhile, rural areas often lack structured physical activity programs, recreational infrastructure, and public awareness regarding healthy lifestyle choices [59]. Socioeconomic constraints may also limit access to nutritious food and exercise opportunities [59]. Thus, children in a rural setting, especially those at the early TTM stages, may be disproportionately affected by environmental and resource limitations.

Our findings have important implications for the design of public health interventions targeting childhood obesity. TTM-based interventions should be stage-specific. For those in the precontemplation and contemplation stages, raising awareness, enhancing motivation, and addressing perceived barriers to exercise are essential. For children in the preparation and action stages, sustained support and accessible resources can help reinforce active behaviors. School-based programs that incorporate comprehensive physical activity curricula—including structured physical education, after-school sports, and active breaks—can foster healthier behaviors. Community-level initiatives to improve access to safe and appealing spaces for physical activity (parks, bike paths) are also vital. Engaging families through education and joint exercise activities can promote a more supportive home environment. Additionally, policies promoting active transportation and urban planning that encourages pedestrian activity can contribute to broader environmental change. Interventions should also be gender- and context-specific. Recognizing the distinct challenges faced by girls and by rural populations can enhance program effectiveness.

This study has several strengths, including its large, diverse sample and longitudinal design, which allowed us to assess the progression of obesity and its association with TTM stages over time. However, several limitations should be acknowledged. First, physical activity and the stage of behavior change were self-reported, which may introduce recall or social desirability bias. Second, the one-year follow-up period, while informative, may not fully capture long-term behavioral and weight trajectories. Third, the TTM framework, while widely used, does not account for all psychosocial influences on behavior change—such as emotional regulation, peer pressure, or mental health. Finally, residual confounding from unmeasured lifestyle variables (diet quality, screen time) cannot be ruled out.

## 5. Conclusions

Our study underscores the critical role of the transtheoretical model’s stages of behavior change in predicting obesity progression among children and adolescents. The elevated risks observed in the precontemplation and contemplation stage, particularly among boys and rural residents, highlight the need for stage-specific and demographically tailored interventions. Incorporating TTM-based strategies into public health programs may enhance the effectiveness of obesity prevention efforts by aligning interventions with individual readiness to change. Future research should explore the long-term effects of stage-tailored interventions and examine additional psychosocial and environmental factors that influence physical activity and obesity outcomes across diverse youth populations.

## Figures and Tables

**Table 1:** Definition of the exercise behavior stages based on TTM.

Exercise Behaviour Stages	Definition
Maintenance stage	I have been exercising for more than 6 months
Precontemplation stage	I don’t plan to start exercising
Contemplation stage	I’m planning to start in the next 6 months
Preparation stage	I’m planning to start in the next 30 days
Action stage	I have been exercising for more less than 6 months

**Table 2:** Distribution of exercise behavior stages and characteristics of the study population stratified by gender groups of the 2019–2020 follow-up study.

Characterictics	Total (n = 5006)	Boys (n = 2501)	Girls (n = 2505)	*p*-Value
Residence				<0.001
Urban	2754 (55.0)	1321 (52.8)	1433 (57.2)	
Rural	2252 (45.0)	1180 (47.2)	1072 (42.8)	
Incidence				<0.001
Overweight	723 (14.3)	438 (17.3)	285 (11.3)	
Obesity	573 (11.3)	309 (12.2)	264 (10.5)	
Transition				<0.001
Overweight	309 (6.2)	172 (6.9)	137 (5.5)	
Obesity	508 (10.1)	267 (10.7)	241 (9.6)	
Exercise behavior stages				<0.001
Maintenance stage	502 (10.0)	224 (9.0)	278 (11.1)	
Precontemplation stage	690 (13.8)	266 (10.6)	424 (16.9)	
Contemplation stage	883 (17.6)	410 (16.4)	473 (18.9)	
Preparation stage	546 (10.9)	286 (11.4)	260 (10.4)	
Action stage	2385 (47.6)	1315 (52.6)	1070 (42.7)	
Specific indicators of exercise behavior stages				
I am currently actively participating in physical exercise	3824 (75.3)	2022 (52.9)	1802 (47.1)	<0.001
I will participate more actively in physical activities in the next six months	2863 (56.4)	1524 (53.2)	1339 (46.8)	<0.001
I currently maintain regular physical activity	3436 (67.7)	1818 (52.9)	1618 (47.1)	<0.001
I have been engaging in regular physical activity for the past six months	4212 (83.0)	2108 (50.0)	2104 (50.0)	<0.001

## Data Availability

The data that support the findings of this study are not publicly available due to privacy reasons but are available from the corresponding author upon request.

## Abbreviations

The following abbreviations are used in this manuscript:

OWOB	Overweight and Obesity
TTM	Transtheoretical Model
PA	Physical Activity
